# Association between healthy eating index-2015 and various cognitive domains in US adults aged 60 years or older: the National Health and Nutrition Examination Survey (NHANES) 2011–2014

**DOI:** 10.1186/s12889-021-11914-2

**Published:** 2021-10-15

**Authors:** Yameng Fan, Yinyin Zhang, Jiaqiao Li, Yamei Liu, Huan Chang, Yude Jiang, Xingxia Tuo, Long Zhou, Yan Yu

**Affiliations:** 1 School of Public Health, Xi’an Jiaotong University, 76 West Yanta Road, Xi’an, Shaanxi 710061 People’s Republic of China; 2 Department of Cardiology, Sichuan Provincial People’s Hospital, University of Electronic Science and Technology of China, Chengdu, China

**Keywords:** Dietary guidance, HEI-2015, Cognitive function, Interaction

## Abstract

**Background:**

Diet, as a modifiable factor, plays an important role in cognitive function. However, the association between adherence to the 2015–2020 Dietary Guidelines for Americans (DGA), measured by Healthy Eating Index (HEI)-2015, and cognitive function remains unclear. This study aims to explore whether HEI-2015 is associated with various cognitive domains and whether such association is modified by age, gender, or ethnicity in the US adults aged 60 years or older using data from the National Health and Nutrition Examination Survey (NHANES) 2011–2014.

**Methods:**

HEI-2015 scores were calculated from 24-h dietary recall interviews. Cognitive function was evaluated by Digit Symbol Substitution Test (DSST, a measure of processing speed), Animal Fluency Test (AFT, a measure of executive function), a subtest from Consortium to Establish a Registry for Alzheimer’s disease (CERAD, a measure of memory), and a composite-z score calculated by summing z scores of individual tests. The associations between HEI-2015 scores and cognitive performance were explored using multiple linear regression models.

**Results:**

A total of 2450 participants aged 60 years or older were included. Participants with higher HEI-2015 scores were more likely to have higher DSST, AFT as well as composite-z scores (*P*<0.05). Significant interaction effects were identified between HEI-2015 and ethnicity in specific cognitive domains (*P*_interaction_<0.05). Among HEI-2015 components, higher intakes of whole fruits and seafood and plant protein were associated with better cognitive performance (*P*<0.05).

**Conclusion:**

Higher adherence to DGA is associated with better cognitive performance, especially regarding processing speed and executive function among the US adults aged 60 years or older.

**Supplementary Information:**

The online version contains supplementary material available at 10.1186/s12889-021-11914-2.

## Background

With the increase in lifespan, and the established association between cognitive decline and age itself across cognitive dimensions, it is becoming increasingly imperative to identify potential preventive behaviors for delaying cognitive decline. On this, diet, as a modifiable factor, is known to play an important role in cognitive function [1]. There is an urgent need for better understanding association between diet and cognition so that strategies can be improved to prevent and to better manage cognitive decline.

While there have been numerous studies examining the association of individual nutrients or foods with cognitive outcomes [2–4], recent approaches emphasize overall diet quality to account for nutrient interactions and reflect real-life dietary behaviors [5, 6]. Diet quality is typically assessed using regional dietary patterns, such as with Mediterranean diet score, or national dietary guidelines, such as with Healthy Eating Index (HEI). As summarized in recent systemic reviews and meta-analyses, increasing evidence suggests that adherence to the Mediterranean diet shows a promising beneficial role on cognitive function [7, 8]. However, regional dietary patterns may not be appropriate in all national contexts. Studies on the associations between adherence to a national dietary guideline and cognitive outcomes are surprisingly sparse and the findings are varied. Some suggest that higher adherence to dietary guideline are associated with better cognitive performance, or reduced risk of cognitive impairment and dementia [9–12], while others are inconclusive [13–16].

Considering the differences in food culture and availability, we adopted the HEI-2015, which is used to assess adherence to the 2015–2020 Dietary Guidelines of Americans (DGA), to reflect the overall diet quality of the US population enrolled in the present study [17]. It was developed by the United States Department of Agriculture (USDA) and National Cancer Institute (NCI) and emphasizes high intake of total vegetables, greens and beans, total fruits, whole fruits, whole grains, dairy, total protein foods, seafood, plant proteins, and fatty acids while limiting the intake of sodium, refined grains, saturated fats, and added sugars. To date, there is no study evaluating the associations between HEI-2015 and multiple domains of cognitive performance in the US population.

Through this research, we want to answer whether aligning with 2015–2020 DGA, measured by HEI-2015, is related to various cognitive domains among US adults aged 60 years or older, and test whether the associations of HEI-2015 with cognitive domains vary by age, gender, or ethnicity using data from the National Health and Nutrition Examination Survey (NHANES) 2011–2014. We hypothesized that higher HEI-2015 scores will be associated with better cognitive performance.

## Methods

### Study population

**Fig. 1:**
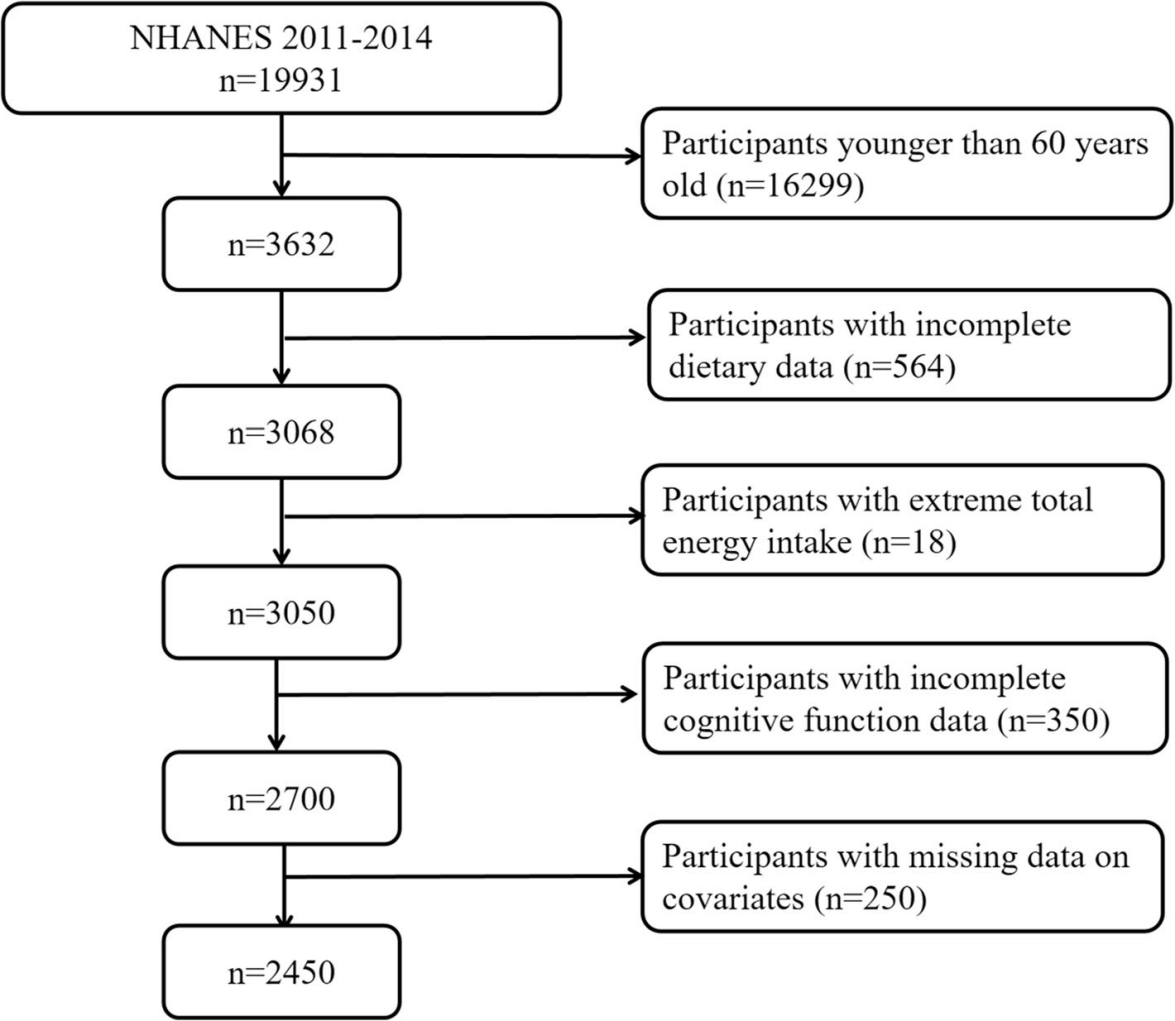
Flow diagram of the study sample selection

The data currently used are publicly available without personally identifiable information from NHANES (https://www.cdc.gov/nchs/nhanes/index.htm). A detailed description of the NHANES study design and methods is available elsewhere [18]. Briefly, NHANES is a program that administers ongoing 2-year-cycle cross-sectional surveys, which is conducted by the Centers for Disease Control and Prevention (CDC). Based on a complex, multistage probability sampling design, NHANES examines a nationally representative sample of ~ 5000 persons each year selected from 15 different locations that are chosen from a sampling frame of all US counties. A major objective of NHANES is to estimate the health and nutritional status of the non-institutional population in the United States. In the present analysis, we combined 2 NHANES cycles (NHANES 2011–2012 and 2013–2014), because these two cycles specifically carried out a series of cognitive function testings. In total, 19,931 individuals participated in NHANES during 2011–2014, and our analysis was limited to 3632 adults aged 60 years or older who were eligible to complete the cognitive function testings. Among them, we excluded individuals with incomplete 24-h dietary recall data (*n* = 564), extreme total energy intake of < 500 or > 5000 kcal/day for females, and < 500 or > 8000 kcal/day for males (*n* = 18), and incomplete cognitive function data (*n* = 350). Moreover, individuals who had missing data on body mass index (BMI), sedentary time, blood pressure (BP), glycohemoglobin (HbA1c), and total cholesterol (TC) were further excluded (*n* = 250). Finally, 2450 individuals were included in our study (Fig. 1). Compared to the original population, study population was more likely to be younger (69.3 years VS 70.1 years), Non-Hispanic white, drinker, to have higher educational level and ratio of family income to poverty. No significant differences were observed in terms of other characteristics (Additional file 1).

### Development of HEI-2015 score

Dietary intake data was collected from NHANES 24-h recall using the USDA automated multiple-pass method [19]. The 24-h dietary recall data were collected for 2 days, which were conducted by a trained interviewer face-to-face in the Mobile Examination Center on one day and a follow-up interview 3–10 days later via phone. Based on the two 24-h dietary recall data, food groups were determined using USDA Food Patterns Equivalence Database, and energy or nutrient content for food and beverage recorded were computed using USDA Food and Nutrient Database for Dietary Studies.

HEI-2015 is a diet quality index developed in partnership by researchers from USDA and NCI to assess the adherence to 2015–2020 DGA and consists of 13 components (food groups or nutrients), including 9 adequacy components (total vegetables, greens and beans, total fruits, whole fruits, whole grains, dairy, total protein foods, seafood and plant proteins, and fatty acids) and 4 moderation components (sodium, refined grains, saturated fats, and added sugars) [20]. These components are scored based on an energy density of 1000 kcal, except for fatty acids which is a ratio of unsaturated to saturated fatty acids. The specific scoring standards for each component are shown in Additional file 2. Briefly, higher intakes result in higher scores for the adequacy components, while lower intakes result in higher scores for moderation components. The component scores are scored separately and summed to compute an overall score with a maximum of 100 (the higher the scores, the higher the compliance with 2015–2020 DGA).

### Assessment of cognitive function

A series of cognitive function testings were performed among adults aged 60 years or older in NHANES 2011–2014, including Digit Symbol Substitution Test (DSST), Animal Fluency Test (AFT), and the word learning subtest from the Consortium to Establish a Registry for Alzheimer’s disease (CERAD). Participants who needed a proxy, did not pass the practice pretest, refused audio record, had communication problems, or gave up will have a missing score for the formal testings. In brief, DSST is used to assess the ability of processing speed [21]. The test provides a paper form that has a key at the top containing 9 numbers paired with symbols. Participants were asked to match corresponding symbols in the 133 boxes that adjoin the numbers in 120 s. The score is the total number of correct matches. AFT is used to evaluate the categorical verbal fluency, a component of executive function, regardless of cultural context [22]. Participants were asked to name as many animals as possible in 60s. A point is given for each named animal, and the final score represents the total number of correct named animals. CERAD word learning subtest was performed to assess both immediate and delayed memory, which consists of three consecutive learning trials and a delayed recall trial [23]. In the learning trials, participants were asked to read aloud 10 irrelevant words. The order of these 10 words is different in each of the three learning trials. After finishing the former two cognitive function testings (DSST and AFT), the delayed recall trial requiring participants to recall 10 words used in the learning trials which were conducted. In addition, a composite-z score was created by summing the z scores [(individual test score - mean score)/SD] of these three individual tests (DSST, AFT, CERAD). For all the tests, higher scores represent better cognitive performance.

### Covariates

The NHANES release information about socio-demographic factors, health-related lifestyles, and health conditions through demographics, questionnaires, examination, and laboratory data. We included some of them as covariates that were thought to be related to cognitive function and/or diet quality based on the previous researches [24, 25]. Socio-demographic factors included age, gender (male and female), ethnicity (Hispanic, non-Hispanic White, non-Hispanic Black, and Asian or other race), and education (less than high school, high school, and more than high school). Additionally, the ratio of family income to poverty was divided into three categories (≤1.30, 1.31–1.85, > 1.85) to reflect house income. The higher ratio indicates better family financial situation. This index was calculated by dividing the family income by the Department of Health and Human Services’ poverty guidelines, specific to family size, survey year and state [26].

As for health-related lifestyles, total time spent sitting except time sleeping (sedentary time) in a day was used as a potential physical activity indicator [27]. Smoker was defined as smoked at least 100 cigarettes in life [28]. Participants who drunk at least 12 times in the previous year were considered drinkers [29].

Concerning health conditions, the depressive symptom was assessed using 9-item Patient Health Questionnaire (PHQ-9). The score of each item ranges from 0 (not at all) to 3 (nearly every day), incorporating a total score with the maximum possible score of 27. Individuals with a PHQ-9 total score of 10 or greater were categorized with depressive symptoms [30]. BMI was calculated as weight (kg) divided by height squared (m) which were measured in Mobile Examination Center. Hypertension was defined as a SBP ≥140 mmHg or a DBP ≥90 mmHg, and/or current use of antihypertensive medication [31]. Hypercholesterolaemia was defined as TC ≥ 240 mg/dl or current use of prescribed medicine for hypercholesterolaemia [32]. Participants were classified as having diabetes based on HbA1c ≥6.5% and/or current use with insulin or diabetic pills [33].

### Statistical analysis

Characteristics of the study population are described as mean ± standard deviation (SD) for continuous variables and number (percentage) for categorical variables. HEI-2015 were categorized based on quartiles (quartile 1: < 25th percentile, quartile 2: ≥25 to 50th percentile, quartile 3: ≥50 to 75th percentile, quartile 4: ≥75th percentile). We tested differences in characteristics between groups with a one-way analysis of variance for continuous variables and with a chi-square test for categorical variables. Linear regression analyses were used to examine the associations between HEI-2015 scores, both as continuous variable and categorical variable (quartiles), and cognitive function**.** To test for trends across increasing quartiles of HEI-2015 scores, the median of HEI-2015 scores in each quartile was calculated and used as a continuous variable. Model 1 was adjusted for age and gender; Model 2 was additionally adjusted for energy intake, ethnicity, BMI, drinking status, smoking status, sedentary time, education, ratio of family income to poverty, depressive symptom, hypertension, hypercholesterolaemia, and diabetes. To explore the dose-response relationship between HEI-2015 and cognitive function, we applied a restricted cubic spline with knots at the 5th, 50th and 95th percentiles of the distribution of the exposure in a fully adjusted model (Model 2) using SAS macro “*%RCS_Reg*” constructed by Desquilbet et al. [34]. We also performed a sensitivity analysis by restricting participants without a depressive symptom. Additionally, stratified analyses were conducted to test whether these associations differ by age groups (60–69 and 70~), gender (male and female), or ethnicity (Hispanic, non-Hispanic white, non-Hispanic black, Asian, or other race). For the exploratory purpose, we further examined the associations between HEI-2015 components (dichotomous) and domain-specific cognitive function adjusting for all covariates and other HEI-2015 components to identify whether statistically significant associations might be attributable to specific components. Data management and dose-response curve were performed using SAS version 9.4 (SAS Institute, Cary, North Carolina, USA) and other statistical analyses were performed using SPSS 24.0 (IBM, Armonk, NY, USA). A two-tailed *P* value < 0.05 was considered statistically significant.

## Results

### Descriptive statistics

The characteristics of the study population according to HEI-2015 quartiles are shown in Table 1. A total of 2450 adults aged 60 years or older were included for analysis. The mean age was 69.3 ± 6.8 years, and 49.8% of the participants were male. Participants with higher adherence to HEI-2015 were more likely to be older, female, Asian or other race, to have lower BMI, higher educational levels, and higher ratio of family income to poverty; while less likely to be smoker, non-Hispanic black race, diabetics, and to have depressive symptom (*P* < 0.05). No significant differences across HEI-2015 quartiles were observed in terms of sedentary time, energy intake, hypertension, and hypercholesterolaemia.

**Table 1:** Characteristics of the overall target population according to HEI-2015 quartiles (*n* = 2450)^1^

	Quartiles of HEI-2015				
Characteristics	Q1^2^	Q2	Q3	Q4	
N	612	613	612	613	*P*^3^
Age (years)	68.5 ± 6.5	69.2 ± 6.8	70.0 ± 6.8	69.8 ± 6.8	<0.001
Gender, n (%)					<0.001
Male	348 (56.9)	327 (53.3)	286 (46.7)	258 (42.1)	
Female	264 (43.1)	286 (46.7)	326 (53.3)	355 (57.9)	
Body mass index (kg/m^2^)	29.8 ± 6.9	29.3 ± 6.1	28.9 ± 5.9	28.3 ± 5.9	<0.001
Ethnicity, n (%)					<0.001
Hispanic	110 (18.0)	120 (19.6)	110 (18.0)	118 (19.2)	
Non-Hispanic white	295 (48.2)	313 (51.1)	305 (49.8)	317 (51.7)	
Non-Hispanic black	174 (28.4)	140 (22.8)	129 (21.1)	108 (17.6)	
Asian or other race	33 (5.4)	40 (6.5)	68 (11.1)	70 (11.4)	
Education, n (%)					<0.001
less than high school	183 (29.9)	153 (25.0)	134 (21.9)	110 (17.9)	
high school	170 (27.8)	159 (25.9)	141 (23.0)	107 (17.5)	
more than high school	259 (42.3)	301 (49.1)	337 (55.1)	396 (64.6)	
Ratio of family income to poverty, n (%)					<0.001
≤ 1.30	205 (33.5)	187 (30.5)	151 (24.7)	135 (22.0)	
1.31 ~ 1.85	126 (20.6)	104 (17.0)	105 (17.2)	106 (17.3)	
>1.85	281 (45.9)	322 (52.5)	356 (58.2)	372 (60.7)	
Energy intake (kcal/d)	1876.8 ± 748.4	1850.2 ± 691.4	1800.7 ± 661.3	1782.0 ± 618.4	0.056
Sedentary time (h/d)	6.6 ± 3.3	6.6 ± 3.1	6.5 ± 3.2	6.4 ± 3.1	0.603
Drinker, n (%)					0.241
Yes	435 (71.1)	440 (71.8)	416 (68.0)	413 (67.4)	
No	177 (28.9)	173 (28.2)	196 (32.0)	200 (32.6)	
Smoker, n (%)					<0.001
Yes	357 (58.3)	336 (54.8)	294 (48.0)	265 (43.2)	
No	255 (41.7)	277 (45.2)	318 (52.0)	348 (56.8)	
Depressive symptom, n (%)					0.012
Yes	61 (10.0)	63 (10.3)	55 (9.0)	34 (5.5)	
No	551 (90.0)	550 (89.7)	557 (91.0)	579 (94.5)	
Hypertension, n (%)					0.339
Yes	427 (69.8)	408 (66.6)	403 (65.8)	400 (65.3)	
No	185 (30.2)	205 (33.4)	209 (34.2)	213 (34.7)	
Diabetes, n (%)					0.045
Yes	180 (29.4)	168 (27.4)	151 (24.7)	140 (22.8)	
No	432 (70.6)	445 (72.6)	461 (75.3)	473 (77.2)	
Hypercholesterolaemia, n (%)					0.903
Yes	294 (48.0)	289 (47.1)	281 (45.9)	290 (47.3)	
No	318 (52.0)	324 (52.9)	331 (54.1)	323 (52.7)	

^1^Values are percentages (%) or means±SD; HEI-2015: Healthy Eating Index-2015

^2^Q, quartiles; Q1 represents the unhealthiest diet quality, Q4 represents the healthiest diet quality

^3^*P*-values refer to differences across tertiles and were calculated using chi-square tests for categorical variables and F-tests for continuous variables

### Association between HEI-2015 score and cognitive function

Table 2 presents the associations between HEI-2015 scores and cognitive performance. In an age and gender-adjusted Model 1, the highest HEI-2015 quartile was significantly associated with higher DSST score (B: 5.26; 95%CI: 3.45, 7.06; *P*<0.001), AFT score (B: 1.31; 95%CI: 0.71, 1.91; *P*<0.001), CERAD score (B: 0.98; 95%CI: 0.30, 1.66; *P*<0.01), and composite z-score for cognitive function (B: 0.70; 95%CI: 0.45, 0.96; *P*<0.001), compared to the lowest HEI-2015 quartile. There was evidence of a linear trend in all of these relations. When treating HEI-2015 as a continuous measure, each 1-unit increase in HEI-2015 score was associated with higher DSST score (B: 0.16; 95%CI: 0.11, 0.21; *P*<0.001), AFT score (B: 0.04; 95%CI: 0.02, 0.05; *P*<0.001), CERAD score (B: 0.03; 95%CI: 0.01, 0.05; *P*<0.01), and composite z-score (B: 0.02; 95%CI: 0.01, 0.03; *P*<0.001). After fully adjusting in Model 2, the associations of HEI-2015 (highest versus lowest) with AFT (B: 0.65; 95%CI: 0.09, 1.21; *P*<0.05) and composite z-scores (B: 0.26; 95%CI: 0.05, 0.47; *P*<0.05) attenuated but remained significant, whereas the associations with DSST and CERAD scores were no longer significant. We observed evidence of a linear trend in these associations of HEI-2015 scores with DSST, AFT and composite z-scores except CERAD. In addition, each 1-unit increase in HEI-2015 was associated with DSST (B: 0.05; 95% CI: 0.01, 0.09; *P*<0.05), AFT (B: 0.02; 95% CI: 0.00, 0.03; *P*<0.01), and composite z-scores (B: 0.01; 95% CI: 0.00, 0.01; *P*<0.01), but not associated with CERAD score.

**Table 2:** Regression coefficients and 95% confidence intervals of HEI-2015 for cognitive function scores (*n* = 2450)^1^

		Cognitive function			
		DSST	AFT	CERAD	Composite z-score^2^
HEI-2015	n	B (95%CI)	B (95%CI)	B (95%CI)	B (95%CI)
Model 1^3^					
Categorical					
Q1^4^	612	0 (Reference)	0 (Reference)	0 (Reference)	0 (Reference)
Q2	613	0.58 (−1.21, 2.38)	0.37 (−0.23, 0.97)	0.08 (−0.60, 0.75)	0.11 (−0.14, 0.37)
Q3	612	2.45 (0.65, 4.25)^**^	0.68 (0.07, 1.28)^*^	0.32 (−0.35, 1.01)	0.32 (0.07, 0.57)^*^
Q4	613	5.26 (3.45, 7.06)^***^	1.31 (0.71, 1.91)^***^	0.98 (0.30, 1.66)^**^	0.70 (0.45, 0.96)^***^
*P*_trend_^5^		<0.001	<0.001	0.004	<0.001
Continuous					
1 unit increase		0.16 (0.11, 0.21)^***^	0.04 (0.02, 0.05)^***^	0.03 (0.01, 0.05)^**^	0.02 (0.01, 0.03)^***^
Model 2					
Q1	612	0 (Reference)	0 (Reference)	0 (Reference)	0 (Reference)
Q2	613	−0.78 (−2.16, 0.60)	0.09 (−0.45, 0.63)	− 0.14 (− 0.78, 0.51)	−0.05 (− 0.26, 0.16)
Q3	612	0.02 (−1.38, 1.42)	0.34 (−0.21, 0.89)	− 0.06 (− 0.71, 0.59)	0.06 (− 0.16, 0.27)
Q4	613	1.40 (− 0.02, 2.82)	0.65 (0.09, 1.21)^*^	0.36 (− 0.30, 1.02)	0.26 (0.05, 0.47)^*^
*P*_trend_		0.031	0.015	0.273	0.011
Continuous					
1 unit increase		0.05 (0.01, 0.09)^*^	0.02 (0.00, 0.03)^**^	0.01 (−0.01, 0.03)	0.01 (0.00, 0.01)^**^

^1^HEI-2015: Healthy Eating Index; B: unstandardized regression coefficient; CI: confidence interval; ^***^P < 0.001, ^**^P < 0.01, ^*^P < 0.05

^2^The composite-z score was calculated by summing the z scores ((test score - mean score)/SD) of the three individual tests

^3^Model 1: adjusted for age and gender; Model 2: Model 1 + daily energy intake, ethnicity, drinking status, smoking status, education, ratio of family income to poverty, sedentary time, depressive symptom, hypertension, hypercholesterolaemia, and diabetes

^4^Q, quartile; Q1 represents the unhealthiest diet quality, Q4 represents the healthiest diet quality

^5^*P*_trend:_ Test for trend based on a variable containing the median value for each quartile

Similar results were observed in sensitivity analyses (Additional file 3). When excluding individuals who suffered a depressive symptom (*n* = 177), HEI-2015 score (as a continuous variable) was still positively associated with DSST (B: 0.05; 95% CI: 0.01, 0.09; *P*<0.01), AFT (B: 0.02; 95% CI: 0.00, 0.03; *P*<0.05) and composite z-scores (B: 0.01; 95% CI: 0.00, 0.01; *P*<0.05) in fully adjusted model.

**Fig. 2:**
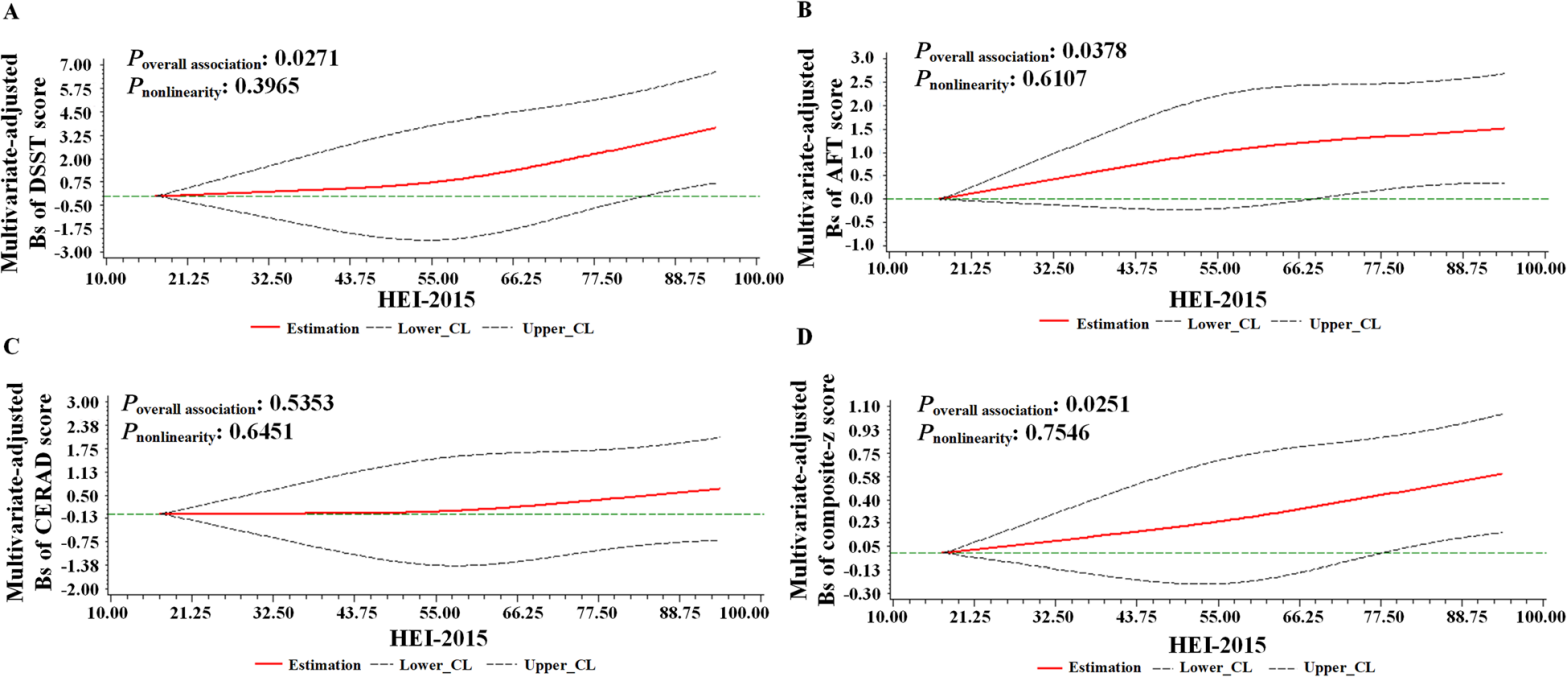
Dose-response relationship of HEI-2015 and various domains of cognitive function. Model is adjusted for age, gender, daily energy intake, ethnicity, body mass index, drinking status, smoking status, education, ratio of family income to poverty, sedentary time, depressive symptom, hypertension, hypercholesterolaemia, and diabetes

The dose-response relationship between HEI-2015 and domain-specific or composite z-score cognitive function after fully adjusting are shown in Fig. 2. In restricted cubic spline models, HEI-2015 scores were positively associated with DSST score (*P*_nonlinearity_ = 0.3965; Fig. 2A), AFT score (*P*_nonlinearity_ = 0.6107; Fig. 2B), and composite z-score (*P*_nonlinearity_ = 0.7546; Fig. 2C) in a linear manner. However, no significant association was observed between HEI-2015 score and CERAD score in the fully adjusted model (Fig. 2D).

### Interaction effects

**Fig. 3:**
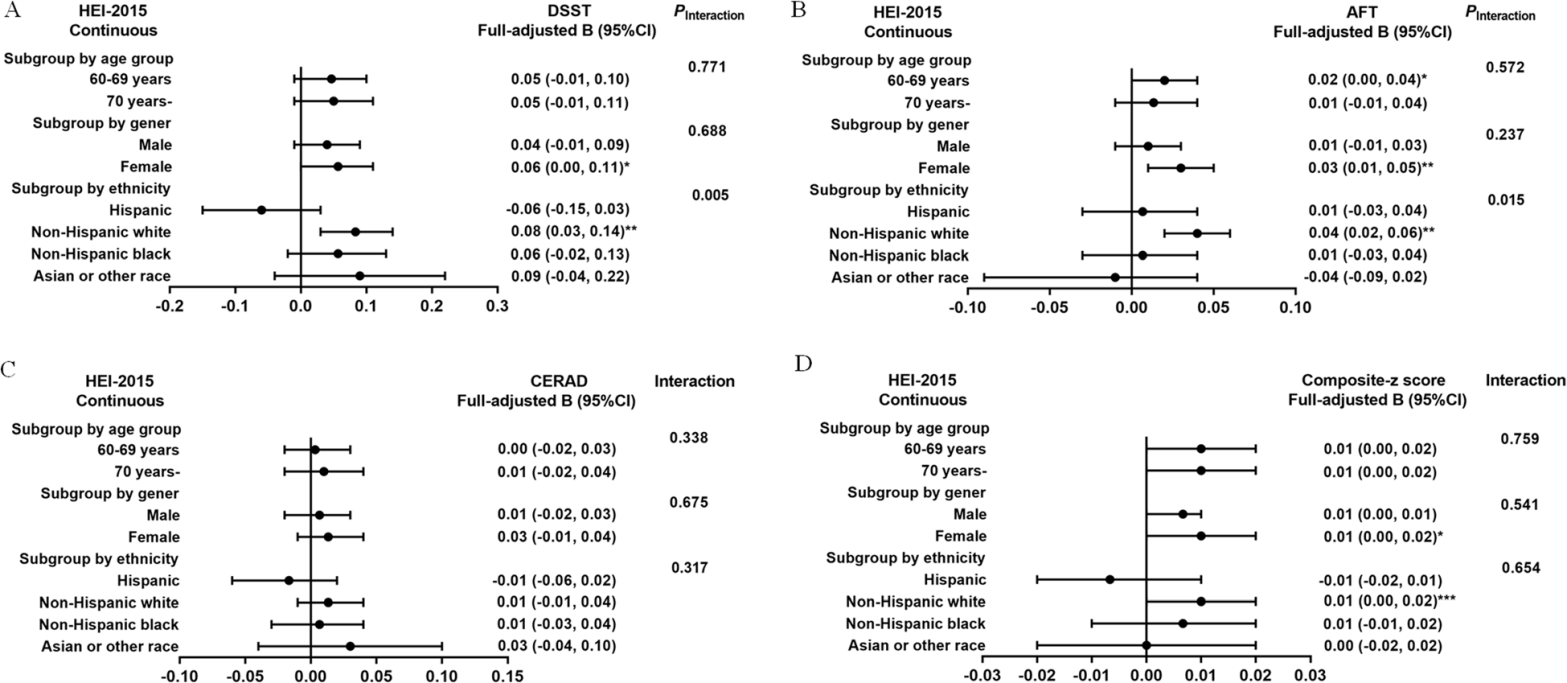
Association between HEI-2015 and various domains of cognitive function stratified by demographic characteristics. Age subgroups are adjusted for age, gender, daily energy intake, ethnicity, body mass index, drinking status, smoking status, education, ratio of family income to poverty, sedentary time, depressive symptom, hypertension, hypercholesterolaemia, and diabetes. Gender subgroups are adjusted for age, daily energy intake, ethnicity, body mass index, drinking status, smoking status, education level, ratio of family income to poverty, sedentary time, depressive symptom, hypertension, hypercholesterolaemia, and diabetes. Ethnicity subgroups are adjusted for age, gender, daily energy intake, body mass index, drinking status, smoking status, education level, ratio of family income to poverty, sedentary time, depressive symptom, hypertension, hypercholesterolaemia, and diabetes. ***P < 0.001, **P < 0.01, *P < 0.05

Potential interaction effects were identified between HEI-2015 (treated as continuous) and ethnicity in relation to DSST score (*P*_interaction_ <0.01; Fig. 3A) and AFT score (*P*_interaction_ <0.05; Fig. 3B). HEI-2015 scores were positively associated with DSST (B: 0.08; 95% CI: 0.03, 0.14; *P*<0.01) and AFT scores (B: 0.04; 95% CI: 0.02, 0.06; *P*<0.01), respectively, among the non-Hispanic white population in the fully adjusted model, but not among Hispanic, non-Hispanic blacks, and Asian or other race. In addition, no interaction effects were observed between age group or gender and HEI-2015 scores (treated as continuous) on any domain-specific cognitive function or composite-z score in the fully adjusted model (*P*_interaction_ > 0.05; Fig. 3A-3D).

### Association between HEI-2015 components and cognitive function

Given the significant associations between higher HEI-2015 total scores and better performance on domain-specific cognitive function assessed by DSST and AFT, an exploratory analysis was performed to test the associations of each HEI-2015 component (dichotomous) with them, adjusting for all covariates included in Model 2 as well as the other components (Additional file 4). Among 13 HEI-2015 components, higher intakes of the whole fruits (B: 1.59; 95% CI: 0.21, 2.98; *P*<0.05), seafood and plant protein (B: 1.46; 95% CI: 0.34, 2.58; *P*<0.05), and fatty acids (B: 1.28; 95% CI: 0.08, 2.48; *P*<0.05) were associated with better performance on DSST. Meanwhile, those with the higher consumption of whole fruits (B: 0.58; 95% CI: 0.03, 1.12; *P*<0.05) and seafood and plant protein (B: 0.78; 95% CI: 0.34, 1.22; *P*<0.001) were more likely to have better performance on AFT.

## Discussion

In this cross-sectional study, we found that adherence to 2015–2020 dietary guidelines for Americans, assessed by HEI-2015, is associated with higher DSST (a measure of processing speed), AFT (a measure of executive function), and composite-z scores in the US adults aged 60 years or older. Notably, the positive associations of HEI-2015 with cognitive performance in the domains of processing speed and executive function are stronger among those of non-Hispanic white background. Additionally, exploratory analysis of components suggests adherence to the recommended intakes of whole fruits and seafood and plant protein were more likely to have better cognitive performance.

Our results are in line with studies that found associations between adherence to national dietary guidelines and cognitive outcomes. Higher adherence to the Dietary Guidelines for Americans, assessed by HEI-2005 or HEI-2010, has been reported to be associated with higher global cognition among Puerto Rican adults living in Boston, and better cognitive performance for White and African Americans from Baltimore, respectively [10, 12]. Likewise, in a middle-aged and elderly Chinese population, the Chinese Dietary Guidelines Index-2018 was significantly associated with a lower risk of mild cognitive impairment [11]. However, our results are inconsistent with the Women’s Health Initiative Memory Study that showed no significant associations between HEI-2010 and cognitive decline in older women [16]. Other studies conducted in Australia, Canada, and Chicago, US also reported that there was no association between adherence to a national dietary guideline and cognitive function [13–15]. These inconsistent results may emerge from variations in HEI components, study populations, or cognitive outcome measures, suggesting more research is needed to clarify the relationship between adherence to dietary patterns and cognitive outcomes. Prior to our study, we only found a relevant analysis from the Atherosclerosis Risk in Communities (ARIC) Study which reported that HEI-2015 was associated with a lower risk of incident dementia in black and white participants from four US communities [9]. Compared with the ARIC, our study enrolled multiethnic populations, assessed multiple cognitive domains, explored the interaction effects between HEI-2015 and some demographic factors, and included two important potential confounding factors: ratio of family income to poverty and depressive symptoms.

In the present study, HEI-2015 scores were not associated with memory sub-domain assessed by CERAD, but were positively associated with processing speed evaluated by DSST and executive function evaluated by AFT in the fully adjusted model, suggesting that some certain healthy dietary pattern may only have benefits for specific cognitive domains. Another analysis in the NHANES population revealed that the Mediterranean diet score was associated with memory and executive function rather than processing speed [35]. An analysis from the Hispanic Community Health Study found that adherence to an alternative to the HEI-2010 was associated with better verbal learning and memory, but not verbal fluency and processing speed [24]. The cognitive performance assessed by multiple cognitive function testings involving different domains may lead to inconsistent results on the relationship between diet and cognitive function. Thus, as many cognitive testings as possible should be used simultaneously to achieve a comprehensive analysis.

Notably, our findings are unique in showing that adherence to dietary guidance, as indicated by HEI-2015, was linked to cognitive performance in non-Hispanic White but not other races. Similar race/ethnicity differences have been observed in previous studies investigating associations between diet and cognition [36]. In the Einstein Aging Study, a healthy diet was related to executive dysfunction specifically in whites [37]. Similarly, the NHANES study showed that a protective role of adherence to the Mediterranean diet on cognition in non-Hispanic White but not in other race/ethnic groups [35]. Current literature cannot clearly explain race/ethnicity differences in the diet-cognition associations. It is possible that statistical power was limited in the smaller sub-sample of minority groups. Among a large sample of middle-aged and older Hispanics/Latinos, an overall healthier diet quality has been found to be associated with better global cognition [24]. Additionally, differences in genetic or environmental risk factors and validity of dietary indices or cognitive testings between racial/ethnic groups may also influence the association between diet and cognition.

Findings from our exploratory analyses suggest that adherence to the recommended intake of the whole fruits were more likely to have better performance on processing speed and executive function. This may be correlated to the protective effects of bioactive substances that are present in fruits, in particular flavonoids, vitamins, and carotenoids, which have anti-oxidant and anti-inflammatory properties [38]. Previous studies have shown that oxidative stress may contribute to the pathogenesis of Alzheimer’s disease [39, 40]. Besides, the benefits of seafood and plant protein on cognition have also been observed in our study. Although it has been reported that higher intakes of seafood and plant protein were associated with better physical performance and lower mortality, there is limited evidence on the relation between seafood and plant protein and cognitive function, and the mechanism requires further study.

The present study has several advantages. First, NHANES enrolled multiethnic population with a relatively large sample size, making it possible to perform subgroup analysis according to age, gender, or ethnicity. To our knowledge, this is the first study to show the associations of HEI-2015 (the latest version of HEI) with various domains of cognitive function among a representative sample of the US population aged 60 years or older. Meanwhile, we conducted a dose-response analysis to assess the association between HEI-2015 score and cognitive performance. Additionally, a sensitivity analysis was performed by excluding participants who suffered depressive symptoms, and the results were stable. However, there are clear limitations that should also be considered. First, as a cross-sectional study design, we cannot ascertain a causal relationship. Second, NHANES did not collect data on cognitive impairment or neurocognitive disorders, thus, we were unable to take this into account when interpreting our findings. Third, the dietary data which was collected from the two days 24 recall, may not be able to reflect the usual intake well. Last but not least, some demographic characteristics of study samples were significantly different (albeit small) in the total samples. Thus, the results may not be generalized to the general population, and should be interpreted with caution.

## Conclusions

We observed that adherence to the 2015–2020 dietary guideline for Americans, assessed by HEI-2015, is associated with cognitive performance, especially regarding processing speed and executive function in a US population aged 60 and over. The positive association between HEI-2015 score and cognitive performance is stronger in non-Hispanic White. Given the rational findings and several limitations in the present study, the results should be further validated in the large prospective cohort study.

## Supplementary Information

**Additional file 1.** Comparison of characteristics between total and included participants.

**Additional file 2.** Components and scoring standards of HEI-2015^1^.

**Additional file 3.** Sensitivity analysis on the associations between HEI-2015 (continuous) and cognition scores when excluding participants with depression symptom (*n* = 2273)^1^.

**Additional file 4.** Regression coefficients and 95% confidence intervals of HEI-2015 components (dichotomous) for DSST and AFT (*n* = 2450)^1^.

## Data Availability

The datasets generated and/or analyzed during the current study are publicly available from the National Health and Nutrition Examination Survey website (https://www.cdc.gov/nchs/nhanes/index.htm).

## Abbreviations

HEI: Healthy Eating Index
NHANES: The National Health and Nutrition Examination Survey
DSST: Digit Symbol Substitution Test
AFT: Animal Fluency Test
CERAD: Consortium to Establish a Registry for Alzheimer’s disease
DGA: Dietary Guidelines for Americans
CDC: Centers for Disease Control and Prevention
USDA: United States Department of Agriculture
NCI: National Cancer Institute
PHQ: Patient Health Questionnaire
BP: Blood pressure
BMI: Body Mass Index
TC: Total cholesterol
