# Supplementary material for: Association between healthy eating index-2015 and various cognitive domains in US adults aged 60 years or older: the National Health and Nutrition Examination Survey (NHANES) 2011–2014
Source: BMC Public Health. 2021 Oct 15;21:1862. doi: 10.1186/s12889-021-11914-2 (PMC8520277; doi:10.1186/s12889-021-11914-2)
Supplement: Supplementary file 1 — Additional file 1. Comparison of characteristics between total and included participants. [file 12889_2021_11914_MOESM1_ESM.docx]

**Additional file 1. Comparison of characteristics between total and included participants**

| Characteristics | NHANES participants aged ≥60 years^1^ | NHANES participants included in this analysis^2^ |  |
| --- | --- | --- | --- |
| n | 3632 | 2450 | *P* |
| Age (years) | 70.1 ± 7.0 | 69.3 ± 6.8 | ＜0.001 |
| Gender, n (%) |  |  | 0.321 |
| Male | 1760 (48.5) | 1219 (49.8) |  |
| Female | 1872 (51.5) | 1231 (50.2) |  |
| Body mass index (kg/m^2^) | 28.9 ± 6.3 | 29.1 ± 6.3 | 0.238 |
| Ethnicity, n (%) |  |  | ＜0.001 |
| Hispanic | 704 (19.4) | 458 (18.7) |  |
| Non-Hispanic white | 1648 (45.4) | 1230 (50.2) |  |
| Non-Hispanic black | 871 (24.0) | 551 (22.5) |  |
| Asian or other race | 409 (11.3) | 221 (8.6) |  |
| Education, n (%) |  |  | ＜0.001 |
| less than high school | 1074 (29.6) | 580 (23.7) |  |
| high school | 844 (23.2) | 577 (23.6) |  |
| more than high school | 1714 (47.2) | 1293 (52.8) |  |
| Ratio of family income to poverty, n (%) |  |  | ＜0.001 |
| ≤1.30 | 1168 (32.2) | 678 (27.7) |  |
| 1.31 ~ 1.85 | 687 (18.9) | 411 (18.0) |  |
| ＞1.85 | 1777 (48.9) | 1331 (54.3) |  |
| Energy intake (kcal/d) | 1802.4 ± 691.6 | 1827.4 ± 682.1 | 0.180 |
| Sedentary time (h/d) | 6.6 ± 3.3 | 6.5 ± 3.2 | 0.407 |
| Drinker, n (%) |  |  | ＜0.001 |
| Yes | 2126 (58.5) | 1704 (69.6) |  |
| No | 1506 (41.5) | 746 (30.4) |  |
| Smoker, n (%) |  |  | 0.354 |
| Yes | 1812 (49.9) | 1252 (51.1) |  |
| No | 1820 (50.1) | 1198 (48.9) |  |
| Depression, n (%) |  |  | 0.352 |
| Yes | 300 (9.4) | 213 (8.7) |  |
| No | 2887 (90.6) | 2237 (91.3) |  |
| Hypertension, n (%) |  |  | 0.426 |
| Yes | 2114 (67.9) | 1638 (66.9) |  |
| No | 1001 (32.1) | 812 (33.1) |  |
| Diabetes, n (%) |  |  | 0.411 |
| Yes | 843 (27.1) | 639 (26.1) |  |
| No | 2272 (72.9) | 1811 (73.9) |  |
| Hypercholesterolaemia, n (%) |  |  | 0.621 |
| Yes | 1627 (52.2) | 1296 (52.9) |  |
| No | 1488 (47.8) | 1154 (47.1) |  |

^1^All participants aged ≥60 years in NHANES.

^2^Participants included in this analysis without missing data on HEI-2015, cognitive testings and other [covariate](D:/Dict/8.9.6.0/resultui/html/index.html#/javascript:;)s.
