# Supplementary material for: Association between healthy eating index-2015 and various cognitive domains in US adults aged 60 years or older: the National Health and Nutrition Examination Survey (NHANES) 2011–2014
Source: BMC Public Health. 2021 Oct 15;21:1862. doi: 10.1186/s12889-021-11914-2 (PMC8520277; doi:10.1186/s12889-021-11914-2)
Supplement: Supplementary file 2 — Additional file 2. Components and scoring standards of HEI-20151. [file 12889_2021_11914_MOESM2_ESM.docx]

**Additional file 2. Components and scoring standards of HEI-2015^1^.**

| Component | Maximum points | Standard for  maximum score | Standard for  minimum score of zero |
| --- | --- | --- | --- |
| Adequacy |  |  |  |
| Total vegetables | 5 | ≥ 1.1 cup equivalents/1,000 kcal | No vegetables |
| Greens and beans | 5 | ≥ 0.2 cup equivalents/1,000 kcal | No Dark Green Vegetables or Legumes |
| Total fruit | 5 | ≥ 0.8 cup equivalents/1,000 kcal | No fruit |
| Whole fruit | 5 | ≥ 0.4 cup equivalents/1,000 kcal | No whole fruit |
| Whole grain | 10 | ≥ 1.5 oz equivalents/1,000 kcal | No whole grain |
| Dairy | 10 | ≥ 1.3 cup equivalents/1,000 kcal | No dairy |
| Total protein foods | 5 | ≥ 2.5 oz equivalents/1,000 kcal | No protein foods |
| Seafood and plant proteins | 5 | ≥ 0.8 cup equivalents/1,000 kcal | No seafood or plant proteins ≤ 1.2 |
| Fatty acid ratio | 10 | (PUFA+MUFA )/SFA ≥ 2.5^2^ | (PUFA+MUFA )/SFA |
| Moderation |  |  |  |
| Sodium | 10 | ≤ 1.1 g/1,000 kcal | ≥ 2.0 g/1,000 kcal |
| Refined grain | 10 | ≤ 1.8 oz equivalents/1,000 kcal | ≥ 4.3 oz equivalents/1,000 kcal |
| Added sugars | 10 | ≤ 6.5% of energy | ≥ 26% of energy |
| Saturated fats | 10 | ≤ 8% of energy | ≥ 16% of energy |

^1^HEI-2015: Healthy Eating Index-2015.

^2^PUFA, poly-unsaturated fatty acid; MUFA, mono-unsaturated fatty acid; SFA, saturated fatty acid.
