# Supplementary material for: Association between healthy eating index-2015 and various cognitive domains in US adults aged 60 years or older: the National Health and Nutrition Examination Survey (NHANES) 2011–2014
Source: BMC Public Health. 2021 Oct 15;21:1862. doi: 10.1186/s12889-021-11914-2 (PMC8520277; doi:10.1186/s12889-021-11914-2)
Supplement: Supplementary file 3 — Additional file 3. Sensitivity analysis on the associations between HEI-2015 (continuous) and cognition scores when excluding participants with depression symptom (n = 2273)1. [file 12889_2021_11914_MOESM3_ESM.docx]

**Additional file 3 Sensitivity analysis on the associations between HEI-2015 (continuous) and cognition scores when excluding participants with depression symptom** **(n = 2273)^1^.**

|  | Cognitive function | | | |
| --- | --- | --- | --- | --- |
|  | DSST | AFT | CERAD | Global cognition^2^ |
| HEI-2015 | B (95%CI) | B (95%CI) | B (95%CI) | B (95%CI) |
| Model 1^3^ | 0.16 (0.11, 0.21)^***^ | 0.03 (0.02, 0.05)^***^ | 0.03 (0.00, 0.05)^**^ | 0.02 (0.01, 0.03)^***^ |
| Model 2 | 0.05 (0.01, 0.09)^**^ | 0.02 (0.00, 0.03)^*^ | 0.01 (-0.01, 0.03) | 0.01 (0.00, 0.01)^*^ |

^1^HEI-2015: Healthy Eating Index; B: unstandardized regression coefficient; CI: confidence interval; ^***^*P*<0.001, ^**^*P* < 0.01, ^*^*P* < 0.05.

^2^The Global cognition score was calculated by summing the z scores ( (test score - mean score)/SD) of the three former individual tests.

^3^Model 1: adjusted for age and gender; Model 2: Model 1 + daily energy intake, ethnicity, drinking status, smoking status, education, ratio of family income to poverty, sedentary time, hypertension, hypercholesterolaemia and diabetes.
