# Supplementary material for: Association between healthy eating index-2015 and various cognitive domains in US adults aged 60 years or older: the National Health and Nutrition Examination Survey (NHANES) 2011–2014
Source: BMC Public Health. 2021 Oct 15;21:1862. doi: 10.1186/s12889-021-11914-2 (PMC8520277; doi:10.1186/s12889-021-11914-2)
Supplement: Supplementary file 4 — Additional file 4. Regression coefficients and 95% confidence intervals of HEI-2015 components (dichotomous) for DSST and AFT (n = 2450)1. [file 12889_2021_11914_MOESM4_ESM.docx]

**Additional file 4. Regression coefficients and 95% confidence intervals of HEI-2015 components (dichotomous) for DSST and AFT (n = 2450)^1^**

|  |  | DSST | AFT |
| --- | --- | --- | --- |
| Components of HEI-2015^2^ | n | B (95%CI) | B (95%CI) |
| Adequacy |  |  |  |
| Total vegetables |  |  |  |
| Low | 1225 | 0(Reference) | 0(Reference) |
| High | 1225 | 0.25 (-0.84, 1.34) | 0.29 (-0.14, 0.71) |
| Greens and beans |  |  |  |
| Low | 1225 | 0(Reference) | 0(Reference) |
| High | 1225 | 0.14 (-0.93, 1.21) | 0.31 (-0.11, 0.73) |
| Total fruit |  |  |  |
| Low | 1225 | 0(Reference) | 0(Reference) |
| High | 1225 | -1.10 (-2.49, 0.28) | -0.54 (-1.08, 0.01) |
| Whole fruits |  |  |  |
| Low | 1225 | 0(Reference) | 0(Reference) |
| High | 1225 | **1.59 (0.21, 2.98)^*^** | **0.58 (0.03, 1.12)^*^** |
| Whole grains |  |  |  |
| Low | 1225 | 0(Reference) | 0(Reference) |
| High | 1225 | 0.33 (-0.70, 1.36) | 0.00 (-0.40, 0.41) |
| Dairy |  |  |  |
| Low | 1225 | 0(Reference) | 0(Reference) |
| High | 1225 | -0.17 (-1.25, 0.90) | 0.10 (-0.33, 0.52) |
| Total protein foods |  |  |  |
| Low | 1225 | 0(Reference) | 0(Reference) |
| High | 1225 | 0.06 (-1.17, 1.28) | 0.06 (-0.42, 0.54) |
| Seafood and plant proteins |  |  |  |
| Low | 1225 | 0(Reference) | 0(Reference) |
| High | 1225 | **1.46 (0.34, 2.58)^*^** | **0.78 (0.34, 1.22)^***^** |
| Fatty acids |  |  |  |
| Low | 1225 | 0(Reference) | 0(Reference) |
| High | 1225 | **1.28 (0.08, 2.48)^*^** | 0.27 (-0.20, 0.74) |
| Moderation |  |  |  |
| Sodium |  |  |  |
| Low | 1225 | 0(Reference) | 0(Reference) |
| High | 1225 | -0.67 (-1.79, 0.45) | 0.24 (-0.20, 0.68) |
| Refined grains |  |  |  |
| Low | 1225 | 0(Reference) | 0(Reference) |
| High | 1225 | 0.25 (-0.82, 1,32) | -0.21 (-0.63, 0.21) |
| Saturated fats |  |  |  |
| Low | 1225 | 0(Reference) | 0(Reference) |
| High | 1225 | -1.13 (-2.31, 0.06) | -0.44 (-0.91, 0.02) |
| Added sugars |  |  |  |
| Low | 1225 | 0(Reference) | 0(Reference) |
| High | 1225 | 0.04 (-1.03, 1.10) | 0.02 (-0.44, 0.40) |

^1^HEI-2015: Healthy Eating Index; B, unstandardized regression coefficient, is adjusted for age, gender, daily energy intake, ethnicity, body mass index, drinking status, smoking status, education, ratio of family income to poverty, sedentary time, depressive symptom, hypertension, hypercholesterolaemia and diabetes; ^***^*P* < 0.001, ^*^*P* < 0.05.

^2^Each component was dichotomized into high group and low group according to the median score, with high group representing better adherence.
